# Serum Metabolites Associated with Blood Pressure in Chronic Kidney Disease Patients

**DOI:** 10.3390/metabo12040281

**Published:** 2022-03-23

**Authors:** Fengyao Yan, Dan-Qian Chen, Jijun Tang, Ying-Yong Zhao, Yan Guo

**Affiliations:** 1 Department of Computer Science, University of South Carolina, Columbia, SC 37027, USA; fyan@email.sc.edu; 2 Faculty of Life Science and Medicine, Northwest University, Xi’an 710127, China; 20193019@nwu.edu.cn; 3 Department of Internal Medicine, University of New Mexico, Albuquerque, NM 87109, USA

**Keywords:** chronic kidney disease, blood pressure, hypertension

## Abstract

Blood pressure is one of the most basic health screenings and it has a complex relationship with chronic kidney disease (CKD). Controlling blood pressure for CKD patients is crucial for curbing kidney function decline and reducing the risk of cardiovascular disease. Two independent CKD cohorts, including matched controls (discovery *n* = 824; validation *n* = 552), were recruited. High-throughput metabolomics was conducted with the patients’ serum samples using mass spectrometry. After controlling for CKD severity and other clinical hypertension risk factors, we identified ten metabolites that have significant associations with blood pressure. The quantitative importance of these metabolites was verified in a fully connected neural network model. Of the ten metabolites, seven have not previously been associated with blood pressure. The metabolites that had the strongest positive association with blood pressure were aspartylglycosamine (*p* = 4.58 × 10^−5^), fructose-1,6-diphosphate (*p* = 1.19 × 10^−4^) and N-Acetylserine (*p* = 3.27 × 10^−4^). Three metabolites that were negatively associated with blood pressure (phosphocreatine, *p* = 6.39 × 10^−3^; dodecanedioic acid, *p* = 0.01; phosphate, *p* = 0.04) have been reported previously to have beneficial effects on hypertension. These results suggest that intake of metabolites as supplements may help to control blood pressure in CKD patients.

## 1. Introduction

Hypertension is the leading risk factor for many diseases, including cardiovascular disease, stroke, kidney disease, etc. [1] One of the diseases studied frequently with hypertension is chronic kidney disease (CKD) [2]. CKD is a major global health concern and carries a heavy economic burden [3]. Hypertension is one of the most common CKD-associated comorbidities. According to statistics from kidney.org, around 50% of CKD patients also suffer from hypertension. Both hypertension and CKD share many common risk factors, such as BMI, age, diabetes, etc. With decline in kidney function, blood pressure is generally increased [4]. The causality between kidney failure and hypertension is not clear and difficult to determine. It is believed that the relationship could be bi-directional, including primary hypertension-induced kidney disease and renal hypertension [5]. Managing hypertension for CKD patients is crucial for reducing cardiovascular disease and related mortality [6]. 

Metabolites are organic compounds, produced intermediately during metabolism. They are used for chemical reactions in cells. Metabolites have been thoroughly studied in CKD. It has been suggested that metabolites have strong clinical value for CKD [7]. Studies have found strong associations between tryptophan metabolites abundance and kidney function decline [8,9], and treatment with 5-methoxytryptophan can ameliorate renal interstitial fibrosis in mouse models [8]. Additionally, some metabolites have also been found to regulate blood pressure. For example, a study found that gut microbial metabolites can regulate blood pressure [10]. Urinary metabolites were found to be associated with blood pressure dependent on a sodium diet in a randomized controlled trial [11]. Another study found that plasma metabolites mediate the association of coarse grain intake with blood pressure in hypertension-free adults [12].

Metabolites have been studied closely with CKD and blood pressure independently, but never in conjunction. In this study, using an approach combining traditional statistical methods and deep learning methods, we show that, within the confinement of CKD, certain metabolites are directly associated with blood pressure after adjusting for CKD severity. 

## 2. Results

Two cohorts with 1376 subjects (discovery: 824; validation: 552) were used for this study. The basic clinical characteristics are summarized in Table 1. The discovery cohort and validation cohort were independently recruited from different hospitals at different time points. We first conducted traditional regression analysis to determine the relationship between basic clinical characteristics and blood pressure (Table 2). CKD stage was found to be marginally associated with blood pressure in the discovery cohort (*p* = 0.06) and the combined dataset (*p* = 0.07), but not in the validation cohort (*p* = 0.6). Sex was not associated with blood pressure. Age, weight, BMI and eGFR were found to be significantly associated with blood pressure in the discovery, validation and combined cohorts. In the combined cohort, weight and BMI had the largest effects on blood pressure (weight, *p* = 1.36 × 10^−7^; BMI, *p* = 5.49 × 10^−7^). These results indicate that increases in age, weight and BMI and decline in kidney function are associated with increase in blood pressure, which is consistent with previous findings. Based on regression results, in the combined cohort, the clinical characteristics together explained 20% variance in blood pressure (adjusted R^2^ = 0.20).

All the subjects’ serum samples went through mass spectrometry to identify metabolites. A total of 25,107 features were identified. To remove collinearity within the features and reduce the dimensionality of the analysis, we performed feature reduction using the correlation clustering algorithm [13], after which 1755 features remained. These features have strong correlations with multiple other features and usually appear as the hub feature in the clusters. Examples of four selected features and their respective clusters can be seen in Supplementary Figure S1. 

**Figure 1:**
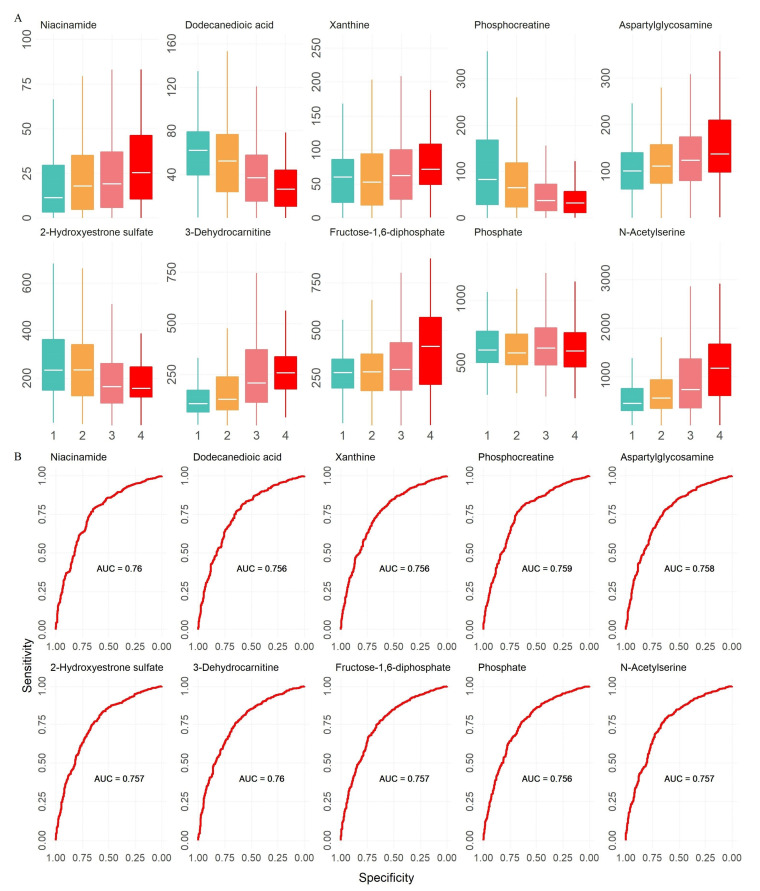
The final ten metabolites that were associated with blood pressure. (**A**). The box plots of the final ten metabolites’ abundances by blood pressure stages (1–4): 1 indicates normal blood pressure, 2 indicates prehypertension, 3 indicates hypertension, 4 indicates crisis. (**B**). The ROC curves of the ten metabolites when treating blood pressure as binary (normal vs. high).

The relationships between these 1755 selected features and blood pressure were evaluated using a linear regression model while adjusting for CKD stage in the discovery cohort, then in the validation cohort. In both cohorts, 31 features were found to be significantly associated with blood pressure, and the exact metabolites for these 31 features were identified. Final regression models were carried out by combining data from both cohorts (Table 3). In the final regression models, we also adjusted for other clinical characteristics that are associated with blood pressure, including age, weight, BMI and eGFR. Ten (six positive, four negative) metabolites remained significantly associated with blood pressure. They were aspartylglycosamine, fructose-1,6-diphosphate, L-glutamic acid, niacinamide, 3-dehydrocarnitine, phosphocreatine, dodecanedioic acid, 2-hydroxyestrone sulfate, xanthine and phosphate. All ten metabolites’ abundances showed substantial trends with blood pressure (Figure 1A). ROC curves showed that all ten metabolites had reasonable areas under the curve when dichotomizing blood pressure into normal and high groups (Figure 1B).

**Figure 2:**
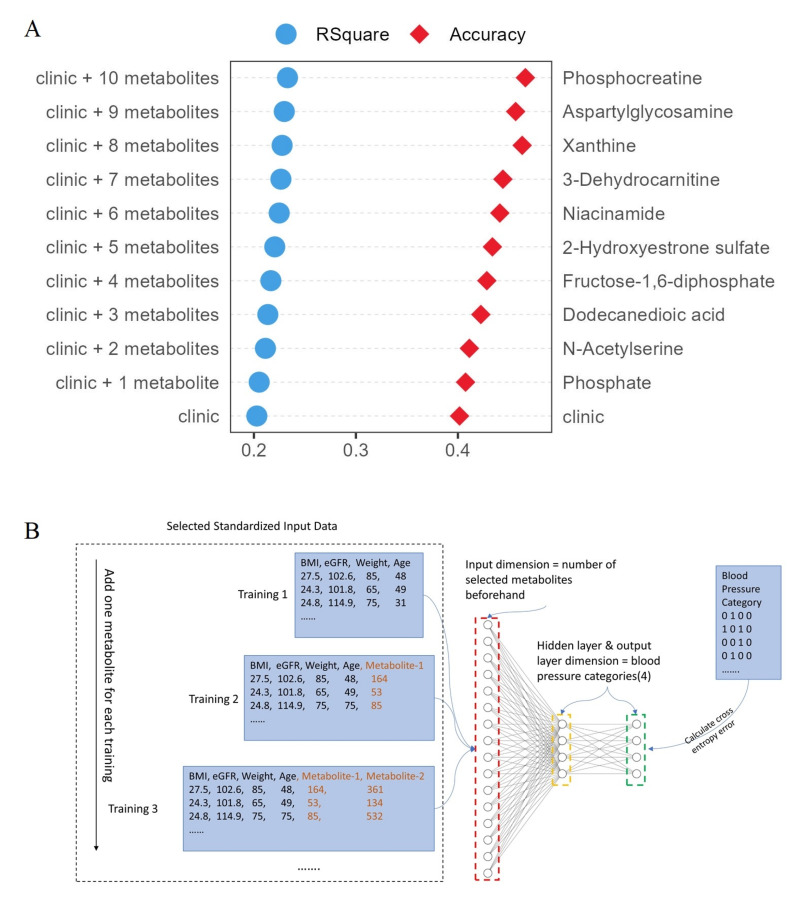
Additional validation of the importance of the final ten metabolites. (**A**) R2 and deep learning model’s accuracy increased as more metabolite was added. (**B**) The fully connected neural network model architecture.

Together, these ten metabolites explained 13.42% variation in blood pressure (adjusted R^2^ = 0.1342). By combining clinical characteristics, the total explainable variance of blood pressure increased to 23.29% (Figure 2A). In addition to adjusted R^2^, we also quantitatively measured the importance of these ten metabolites using deep learning models. A baseline deep learning model was constructed using fully connected neural networks with traditional hypertension risk factors (age, weight, BMI, eGFR). This model had a prediction accuracy of 46%, which was better than random chance (20%, as there are four blood pressure stages) but far from being suitable for application in a clinical setting. By adding the additional ten metabolites, the model’s accuracy increased to 52% (Figure 2A). Note that the goal of constructing this model was not to predict blood pressure based on clinical factors or metabolites; it was, rather, to provide an alternative quantitative measurement of the added importance of the metabolites. 

## 3. Discussion

It is estimated that around 20% of the world’s adults have hypertension. Hypertension is closely intertwined with many diseases, including CKD, which has an estimated global burden of 10% (www.kidney.org, accessed on 16 January 2022). Hypertension is both a cause and a consequence of CKD, making controlling blood pressure a vital strategy to slow down CKD progression [14] and thus reduce the risk of cardiovascular diseases [15]. Recently, it has been argued that metabolic dysfunction underlies essential hypertension [16]. The associations between metabolites and blood pressure have been demonstrated strongly [17,18]. 

We designed a study to examine the relationship between metabolites and blood pressure within the context of CKD. Two large cohorts (discovery and validation) of 1376 subjects were recruited. Metabolites were detected in the patients’ serum samples using mass spectrometry. Through a series of statistical analyses, we identified ten metabolites associated with blood pressure after adjusting for CKD severity. The importance of these ten metabolites was further validated through statistical analysis of R^2^ and accuracy analysis from the fully connected neural network model. Of the ten identified metabolites, many were novel and have not been found to affect blood pressure. For example, the metabolite that had the most significant association with blood pressure was aspartylglycosamine (combined cohort, *p* = 4.58 × 10^−5^). Aspartylglycosamine was firstly identified as a biomarker of hypertension under CKD and also as a biomarker of a congenital disorder of deglycosylation [19,20]. There was evidence that several identified metabolites had connections with blood pressure. We found that fructose 1,6-diphosphate was negatively associated with blood pressure (combined cohort, *p* = 1.19 × 10^−4^). As a glycolytic intermediate, fructose 1,6-diphosphate treatment mitigated ischemic acute renal failure [21] and prevented alcoholic liver disease [22]. Even though no direct evidence indicated the interaction between fructose 1,6-diphosphate and blood pressure, fructose 1,6-diphosphate is involved in pulmonary artery pressure [23] and has protective effect against cardiovascular diseases [24], which may explain the negative association with blood pressure. 

Phosphocreatine was negatively associated with blood pressure (combined cohort, *p* = 6.39 × 10^−3^), which had similar trends with a previous study in cardiovascular disease [25]. Treatment with phosphocreatine prevented cardiovascular disease through modulating the creatine kinase/phosphocreatine energy buffer and transport system [26,27] and inhibited kidney injury via the regulation of the ERK/Nrf2/HO-1 signaling pathway [28]. Dodecanedioic acid is a dicarboxylic acid and is involved in a metabolic pathway intermediate between those of lipids and carbohydrates. Our results show that it was negatively associated with blood pressure (combined cohort, *p* = 0.01). Although no direct association with blood pressure has been found, dodecanedioic acid was found to help maintain blood sugar levels [29] and reduce muscle fatigue [30] in diabetic patients. 

Some identified metabolites were closely related to hypertension or blood pressure regulation. Notably, the tryptophan metabolic pathway was deeply involved in hypertension, including kynuramine and 5-hydroxytryptamine, identified in this study. Our results indicated a negative association between kynuramine and hypertension and a positive association between 5-hydroxytryptamine and hypertension. A previous study proved that treatment with 5-hydroxytryptamine lowered blood pressure in normotensive and hypertensive subjects through the 5-hydroxytryptamine _7_ receptor [31,32].

Additionally, in the present study, we identified that a backbone of mammalian cell membranes, sphingosine phosphate, was negatively associated with blood pressure. A number of studies have confirmed the protective effects of sphingosine phosphate in hypertension and blood pressure regulation, indicating that the modulation of sphingosine phosphate signaling is a potential therapeutic target of hypertension under CKD [33,34,35,36], which was consistent with our results. Serum 3-methyladenine was not found to be associated with blood pressure in the combined cohort (combined cohort, *p* = 0.6). A previous study showed trends of 3-methyladenine levels consistent with our study of hypertension. After treatment with 3-methyladenine, vascular smooth muscle cells were sensitive to senescence that contributed to hypertension, indicating 3-methyladenine as a potential risk factor for hypertension [37]. Here, we found that *N*-acetylneuraminic acid was negatively associated with blood pressure. However, a previous study showed that *N*-acetylneuraminic acid was positively associated with hypertension in patients with cardiovascular disease, which may be a result of different pathological states [38]. Our study excluded diabetic patients, but the negative association with blood pressure may be related to the previous findings. Phosphate is an anion, salt, functional group derived from phosphoric acid. There have been some findings regarding phosphate and blood pressure. One study found that a high intake of phosphate increased blood pressure in young adults [39]. However, another study found that increased phosphate intake as a supplement can serve as a preventive measure for hypertension [40]. In our study, the average age of participants was over 55, and phosphate was negatively associated with blood pressure (combined cohort, *p* = 0.04), which is more concordant with the second study. 

## 4. Materials and Methods

### 4.1. Study Cohorts

Our study contained two large cohorts of CKD patients and matched controls. All participants were ethnically Chinese, and all patients provided written informed consent. The first cohort contained 824 subjects (control = 114, CKD1 = 125, CKD2 = 133, CKD3 = 131, CKD4 = 150, CKD5 = 141) who were recruited between February 2011 and 2013 from the Affiliated Hospital of Shaanxi Institute of Traditional Chinese Medicine. The second cohort contained 552 subjects (control = 96, CKD1 = 97, CKD2 = 76, CKD3 = 94, CKD4 = 93, CKD5 = 196) who were recruited between 2013 and 2016 from Xi’an No. 4 Hospital and Baoji Central Hospital. The four-variable equation of the Modification of Diet in Renal Disease (MDRD) Study was used to estimate GFR (eGFR) [41]. Patients were classified into CKD stages one to five based on CREA-based eGFR equations [42]. Subjects with liver disease, active vasculitis, gastrointestinal pathology or acute kidney diseases were excluded. Health controls were selected based on the following exclusion criteria: history of kidney disease, cardiovascular disease, hypertension, diabetes. The study was approved by the Ethical Committee. We denote the first cohort as the discovery cohort and the second cohort as the validation cohort.

### 4.2. Blood Pressure

Blood pressure was classified into five groups based on conventional definition: normal (systolic < 120 and diastolic < 80), elevated (systolic: 120–129 and diastolic < 80), high stage 1 (systolic: 130–139 and diastolic: 80–89), high stage 2 (systolic: 140–180 or diastolic: 90–120) and hypertensive (systolic > 180 or diastolic > 120). Blood pressure was subsequently coded numerically as 1 to 5 for further analysis. 

### 4.3. High-Throughput Metabolomics and Assessment

Serum samples were collected from all participants. Serum samples were obtained after overnight fasting and sera were separated and stored at −80 °C for biochemical analysis. Blood biochemistry was determined by the clinical laboratory. The metabolomic procedure included sample preparation, metabolite separation and detection. Data preprocessing and statistical analysis for metabolite identification was performed following our previously described protocol [8]. The serum samples were analyzed using a Waters Acquity™ UPLC system equipped with a Waters Xevo™ G2 QTof MS (Milford, MA, USA). Serum samples were separated at 45 °C by an Acquity UPLC HSS T3 column (2.1 × 100 mm, 1.8 μm, Waters, MA, USA). The mobile phases were water (A) and acetonitrile (B) with 0.1% formic acid at the flow rate of 0.45 mL/min. The gradient program was optimized as follows: 0–0.5 min, 1% B; 0.5–12.0 min, 1–30% B; 12.0–15.0 min, 30–99% B; 15.0–16.0 min, 99% B; 16.0–20.0 min, 99.0–1.0% B. The 2 μL sample solution was injected for each run at 4 °C. 

Mass spectrometry was performed with a Waters XevoTM G2 QTof MS. The scan range was from 50 to 1200 m/z. For positive and negative ESI modes, the capillary and cone voltages were set at 2.5 kV and 45 V, respectively. The desolvation gas was set at 550 °C with a desolvation gas rate of 900 L/h. The source temperature was set at 120 °C, and the cone gas rate was set as 50 L/h. The data were obtained in centroid mode. The LockSpray frequency was set as 10 s, and the data were averaged over 10 scans. The data acquisition rate was set to 0.1 s with a 0.1 s interscan delay. During analysis, centroid data were collected with a scan time of 0.1 s and an interscan delay of 0.02 s. Leucine–enkephalin was used as the lockmass at the level of 300 ng/mL with a flow rate of 5 μL/min.

To obtain a pooled quality control sample, 50 µL of all the samples were pooled, and 10 ions, including m/z 161.9852, 391.2812, 381.2846, 764.5300, 441.1958, 820.8169, 208.1360, 133.0861, 486.2534 and 429.2741, were extracted for assessment. The continuous analyses of 6 replicates of quality control samples were used to measure injection precision. RSD% of retention times and peak areas were also measured using quality control samples. The sample preparation repeatability was calculated by 6 parallel samples, and the method repeatability of RSD% of retention times and peak areas of 10 ions was measured from quality control samples. The above-mentioned procedure was performed every day.

### 4.4. Feature Reduction

Overall, 25,107 features were identified through mass spectrometry. Each feature was described by its retention time and molecular weight. Many of the features were highly correlated. We conducted feature reduction using a correlation clustering algorithm (Algorithm 1) [13]. The algorithm works as follows:
**Algorithm 1** Correlation Clustering AlgorithmRequire: *V, E*^+^*, E*^−^    *H* ← ∅  while *V* ≠ ∅ do   *C* ← ∅   *V’* ← ∅   Pick a random pivot *i* ∈ *V*   *C* ← {*i*}   for *j* ∈ *V* and *j* ≠ *i* do    if *j, i* ∈ *E*^+^ then     Add *j* to *C*    end if     if *j, i* ∈ *E*^−^ then     Add *j* to *V’*    end if   end for   *V* ← *V’*   Add *C* to *H*
  end while  return *H*

V is a collection of features, $E^{+}$ is a positive collinearity table and $E^{-}$ is a negative collinearity table. H is a collection of found collinearity clusters. A collinearity cluster contains multiple features that have high collinearity between each other. To summarize Algorithm 1, a random pivot is selected from the collection of features, and a group of features highly collinear to the pivot is stripped away from the collection and forms a new cluster. The process repeats until the collection is exhausted. To check whether a feature pair i,j is in $E^{+}$ or $E^{-}$ from Algorithm 1, we used a special collinearity check procedure (Algorithm 2) as follows:
**Algorithm 2** Pearson Correlation Threshold CheckRequire: *i, j, t*  *P* ← *pearson*(*i, j*)  *Q* ← |*P*|  return *Q > t*

i, j, t are the pivot feature, a feature and a threshold, respectively. The threshold t is a scalar and is set to decide whether to accept a collinearity score. If accepted, $i,j\in E^{+}$, otherwise $i,j\in E^{-}$.The absolute values of Pearson correlation coefficients were used during feature reduction to ignore correlation direction. Threshold t was set to be 0.45. This algorithm will generate a collection of clusters. High collinearity between features presents in each cluster; thus, the cluster can be replaced with one feature selected from the cluster. A specific feature can then be selected from each cluster as needed.

### 4.5. Statistical Analysis

In the discovery cohort, linear regression was carried out to evaluate the relationship between blood pressure and metabolites. To account for the potential correlation between metabolites and CKD stages, an interaction term between feature and CKD stage was added to the regression model. Statistically significant features recurrent in the model of the validation cohort were retained. A final regression model was conducted combining samples from both cohorts. The Benjamini–Hochberg method was used as the multiple test correction method.

### 4.6. Deep Learning Analysis

A feature importance algorithm [43] was used to evaluate the additive importance of the features selected from the algorithm. Firstly, the selected features and data were standardized, and the categorical blood pressure data were further encoded using one-hot encoding (with scikit-learn packages). Secondly, a model was constructed based on fully connected neural networks [44] (with the Keras package in Python) using the blood pressure associated features. Then, each feature was removed from the model and change of model performance was recorded. A decrease in accuracy indicated positive importance; an increase in accuracy indicated negative importance. The magnitude of accuracy alternation served as a quantitative measurement of feature importance.

### 4.7. Metabolites Identification

The final list of blood pressure-associated metabolites was identified and annotated using exact molecular weights, m/z element composition using MassLynx i-FIT software (Waters Corporation, Milford, MA, USA), MS, MS^E^ fragment, literature comparisons and database searches, including the Human Metabolome Database (http://www.hmdb.ca, 5 October 2021), KEGG (http://www.kegg.com, 5 October 2021), METLIN (https://metlin.scripps.edu, 5 October 2022), MassBank (https://massbank.eu/MassBank/, 5 October 2021) and Chemspider (http://www.chemspider.com/, 5 October 2021). Additionally, some metabolites were confirmed by comparison with available reference standards under the same UPLC–HDMS condition.

### 4.8. Study Approval

This study was approved by the Ethical Committee of Shaanxi Traditional Chinese Medicine Hospital (permit number: SXSY-235610), and written informed consent was received from all participants before inclusion in the study. The clinical investigation was conducted according to the principles expressed in the Declaration of Helsinki.

## 5. Conclusions

By conducting a large-scale high-throughput metabolomics study, we identified ten metabolites that were associated with blood pressure after adjusting for the severity of CKD and other hypertension risk factors. With clinical characteristics and metabolites, we explained 23.29% of the variance in blood pressure. A deep learning model that utilized both clinical characteristics and metabolites reached an accuracy of 52%. Although better than random chance, these results are not clinically meaningful and further demonstrate that hypertension is a complex disease with many potential risk factors. The identified metabolites potentially hint at alternative metabolomics-based strategies for controlling hypertension in CKD patients. 

## Figures and Tables

**Table 1:** Sample descriptions and basic clinical measurements.

Dataset	Clinical Characteristics	Normal	CKD1	CKD2	CKD3	CKD4	CKD5
Discovery	Sample Size	144	125	133	131	150	141
	Men (%)	62.50%	45.60%	57.10%	58.80%	54.70%	48.90%
	Age (years)	57.28 ± 17.66	54.65 ± 8.54	56.41 ± 10.2	55.36 ± 15.44	59.51 ± 14.27	59.86 ± 16.41
	eGFR	107.03 ± 15.73	109.75 ± 16.48	78.95 ± 12.32	44.53 ± 11.75	21.7 ± 4.95	8.18 ± 3.07
	Weight	70.36 ± 11.9	69.18 ± 12.83	73.13 ± 11.13	74.08 ± 12.08	73.07 ± 13.29	72.41 ± 13.1
	BMI	24.34 ± 3.36	24.39 ± 3.49	23.78 ± 3.08	24.09 ± 3.39	24.68 ± 3.11	25.58 ± 3.28
	Systolic pressure	124.93 ± 17.65	127.19 ± 19.86	127.99 ± 15.44	146.52 ± 25.25	142.75 ± 20.42	146.48 ± 20.77
	Diastolic pressure	77.6 ± 11.67	79.18 ± 12.64	80.63 ± 11.87	89 ± 16.9	77.97 ± 13.78	81.56 ± 15.62
Validation	Sample Size	96	97	76	94	93	96
	Men (%)	61.50%	52.60%	56.60%	61.70%	54.80%	52.10%
	Age (years)	57.74 ± 15.69	55.94 ± 7.79	52.78 ± 9.18	57.62 ± 14.64	59.05 ± 14.45	58.56 ± 14.5
	eGFR	106.06 ± 11.64	106.39 ± 11.53	78.54 ± 10.72	44.48 ± 13.26	21.55 ± 4.54	8.77 ± 3
	Weight	69.79 ± 11.41	71.21 ± 13.14	73.53 ± 11.95	71.9 ± 12.76	72.48 ± 11.56	72.54 ± 12.61
	BMI	24.18 ± 3.19	24.68 ± 3.25	23.6 ± 3.11	24.8 ± 3.5	25.27 ± 3.63	25.76 ± 3.47
	Systolic pressure	125.12 ± 19.72	127.1 ± 17.99	127.76 ± 16.73	145.18 ± 27.47	140.94 ± 21.44	149.59 ± 20.78
	Diastolic pressure	78.26 ± 13.11	78.33 ± 12.16	80.51 ± 12.83	85.4 ± 15.49	76.44 ± 11.37	83.64 ± 14.71

**Table 2:** Associations between clinical characteristics and blood pressure.

Cohort	Clinical Characteristics	Estimate ^1^	Stderr ^2^	*p* ^3^
Discovery (*n* = 824)	(Intercept)	1.5874	0.3458	5.11 × 10^−6^
	CKD	0.0819	0.0443	6.49 × 10^−2^
	eGFR	−0.0042	0.0019	2.56 × 10^−2^
	Sex	0.0667	0.0530	2.09 × 10^−1^
	Age	0.0037	0.0019	4.88 × 10^−2^
	Weight	0.0095	0.0022	1.32 × 10^−5^
	BMI	0.0337	0.0082	4.42 × 10^−5^
Validation (*n* = 552)	(Intercept)	1.8901	0.4203	8.44 × 10^−6^
	CKD	0.0284	0.0601	6.37 × 10^−1^
	eGFR	−0.0062	0.0026	1.62 × 10^−2^
	Sex	0.0624	0.0657	3.43 × 10^−1^
	Age	0.0061	0.0025	1.44 × 10^−2^
	Weight	0.0082	0.0028	3.17 × 10^−3^
	BMI	0.0300	0.0100	2.81 × 10^−3^
Combined (*n* = 1376)	(Intercept)	1.7042	0.2657	1.94 × 10^−10^
	CKD	0.0631	0.0355	7.55 × 10^−2^
	eGFR	−0.0049	0.0015	1.27 × 10^−3^
	Sex	0.0660	0.0411	1.09 × 10^−1^
	Age	0.0045	0.0015	2.44 × 10^−3^
	Weight	0.0090	0.0017	1.36 × 10^−7^
	BMI	0.0320	0.0063	4.59 × 10^−7^

^1^ Estimate (effect size) from linear regression with blood pressure as outcome. A positive estimate indicates a positive association; a negative estimate indicates a negative association.^2^ Standard error from the linear regression model. ^3^ *p*-value from the linear regression model.

**Table 3:** Linear regression results showing associations between metabolites and blood pressure.

				Discovery		Validation		Combined		
Metabolites	Identification Confidence ^a^	MS	Retention Time	Estimate ^b^	*p* ^c^	Estimate ^b^	*p* ^c^	Estimate ^b^	*p* ^c^	Adjusted *p* ^d^
Aspartylglycosamine	EC, MS, MS^E^, database	412.0567	4.53	0.0015	1.44 × 10^−2^	0.002	5.63 × 10^−3^	0.0007	4.58 × 10^−5^	1.42 × 10^−3^
Fructose-1,6-diphosphate	EC, MS, MS^E^, database	447.9958	2.89	0.0008	3.61 × 10^−2^	0.001	4.48 × 10^−2^	0.0004	1.19 × 10^−4^	1.84 × 10^−3^
L-Glutamic acid	Reference standard	226.074	0.98	0.0004	1.36 × 10^−3^	0.0003	4.27 × 10^−2^	0.0001	3.27 × 10^−4^	3.38 × 10^−3^
Niacinamide	EC, MS, MS^E^, database	283.0602	3.44	0.0028	1.32 × 10^−2^	0.0077	1.09 × 10^−4^	0.0017	5.30 × 10^−4^	4.11 × 10^−3^
3-Dehydrocarnitine	EC, MS, MS^E^, database	236.0078	3.57	0.0011	2.17 × 10^−2^	0.0014	1.84 × 10^−2^	0.0004	1.27 × 10^−3^	7.87 × 10^−3^
Phosphocreatine	Reference standard	294.0924	5.14	−0.0012	4.44 × 10^−2^	−0.0015	1.24 × 10^−2^	−0.0006	6.39 × 10^−3^	3.30 × 10^−2^
Dodecanedioic acid	Reference standard	269.1144	3.47	−0.0035	1.79 × 10^−2^	−0.0046	2.64 × 10^−2^	−0.0011	1.38 × 10^−2^	6.11 × 10^−2^
2-Hydroxyestrone sulfate	EC, MS, MS^E^, database	408.1494	3.01	−0.0006	4.72 × 10^−2^	−0.001	2.20 × 10^−2^	−0.0003	2.07 × 10^−2^	8.02 × 10^−2^
Xanthine	Reference standard	368.0554	3.86	0.0013	2.51 × 10^−2^	0.0016	3.19 × 10^−2^	0.0003	4.01 × 10^−2^	1.38 × 10^−1^
Phosphate	EC, MS, MS^E^, database	181.0374	0.87	−0.0003	1.03 × 10^−2^	−0.0003	4.94 × 10^−2^	−0.0001	4.56 × 10^−2^	1.41 × 10^−1^
NADP^+^	EC, MS, MS^E^, database	391.0605	4.09	0.0004	5.68 × 10^−3^	0.0003	2.39 × 10^−2^	0	8.34 × 10^−2^	2.24 × 10^−1^
Coenzyme A	Reference standard	393.0802	4.33	0.0002	2.99 × 10^−2^	0.0002	7.57 × 10^−3^	0	8.69 × 10^−2^	2.24 × 10^−1^
Nicotine glucuronide	EC, MS, MS^E^, database	415.0683	0.87	0.0005	1.40 × 10^−2^	−0.0005	2.10 × 10^−2^	−0.0001	1.12 × 10^−1^	2.55 × 10^−1^
Dihydroasparagusic acid	EC, MS, MS^E^, database	305.0033	1.34	0.0029	2.08 × 10^−4^	0.0036	1.67 × 10^−4^	0.0003	1.15 × 10^−1^	2.55 × 10^−1^
N2-Methylguanine	EC, MS, MS^E^, database	210.0359	5.48	0.0061	1.78 × 10^−4^	0.0048	1.29 × 10^−2^	0.0003	1.25 × 10^−1^	2.58 × 10^−1^
Butyl acetate	Reference standard	81.0702	4.96	0.0131	3.78 × 10^−2^	−0.0088	4.61 × 10^−2^	−0.0026	1.51 × 10^−1^	2.93 × 10^−1^
Kynuramine	Reference standard	392.2132	3.8	−0.0004	3.72 × 10^−2^	−0.0005	3.93 × 10^−2^	−0.0001	1.75 × 10^−1^	3.19 × 10 ^−1^
N-Myristoyl Alanine	EC, MS, MS^E^, database	306.2628	4.67	0.007	4.45 × 10^−2^	0.0091	2.23 × 10^−2^	−0.0002	2.09 × 10^−1^	3.60 × 10^−1^
N-Acetylputrescine	Reference standard	207.0297	0.85	0.0047	2.64 × 10^−3^	0.0052	1.26 × 10^−2^	0.0003	2.65 × 10^−1^	4.32 × 10^−1^
Undecanedioic acid	EC, MS, MS^E^, database	199.136	4.75	0.0028	8.12 × 10^−3^	0.0034	2.63 × 10^−2^	0.0004	3.28 × 10^−1^	5.00 × 10^−1^
dUDP	EC, MS, MS^E^, database	206.0009	4.65	0.0029	3.64 × 10^−2^	−0.0045	1.98 × 10^−2^	0.0005	3.39 × 10^−1^	5.00 × 10^−1^
5-Hydroxytryptamine	Reference standard	177.1022	3.18	0	1.63 × 10^−2^	0	1.76 × 10^−2^	0	4.39 × 10^−1^	6.00 × 10^−1^
Methionine sulfoxide	EC, MS, MS^E^, database	244.065	2.42	0.0103	2.80 × 10^−3^	0.0131	1.04 × 10^−3^	0	4.45 × 10^−1^	6.00 × 10^−1^
Selenocysteine	EC, MS, MS^E^, database	355.9162	2.93	−0.0018	2.56 × 10^−2^	−0.0028	1.38 × 10^−2^	−0.0002	5.33 × 10^−1^	6.88 × 10^−1^
N-Acetylneuraminic acid	Reference standard	332.0959	0.98	−0.0285	4.95 × 10^−2^	−0.0483	4.48 × 10^−2^	0	5.90 × 10^−1^	7.00 × 10^−1^
N-Acetylgalactosamine 6-sulfate	EC, MS, MS^E^, database	365.0669	2.73	0.0085	1.47 × 10^−2^	0.0088	3.97 × 10^−2^	0.0001	6.04 × 10^−1^	7.00 × 10^−1^
3-Methyladenine	Reference standard	172.0588	2.89	0.0016	9.56 × 10^−3^	0.0018	2.67 × 10^−2^	0	6.10 × 10^−1^	7.00 × 10^−1^
N-Acetylaspartylglutamic acid	EC, MS, MS^E^, database	322.1209	4.21	−0.0004	1.90x10^−2^	0.0005	4.01 × 10^−2^	0	7.17 × 10^−1^	7.71 × 10^−1^
Sphingosine-1-phosphate	EC, MS, MS^E^, database	356.1989	5.44	−0.0015	3.84 × 10^−2^	0.0019	2.80 × 10^−2^	0.0001	7.21 × 10^−1^	7.71 × 10^−1^
Oxodecanoylcarnitine	EC, MS, MS^E^, database	271.1503	4.52	0.0004	3.69 × 10^−2^	−0.0006	4.41 × 10^−2^	0	8.79 × 10^−1^	8.88 × 10 ^−1^
2-Methylguanosine	EC, MS, MS^E^, database	342.0801	3.26	−0.0004	2.41 × 10^−2^	0.0002	4.79 × 10^−2^	0	8.88 × 10^−1^	8.88 × 10^−1^

^a^ Metabolites were identified and their identities confirmed by a pure substance. Other metabolites were annotated based on elemental composition, MS, MS^E^ and by comparison with reference libraries. EC, elemental composition. ^b^ Estimate (effect size) from linear regression with blood pressure as outcome. A positive estimate indicates a positive association; a negative estimate indicates a negative association. ^c^ *p*-values from linear regression models. ^d^ Adjusted *p*-value according to the Benjamini–Hochberg method.

## Data Availability

The data are available upon a collaborative request. The data are not publicly available due to restrictions of the original IRB and consent.

## Supplementary Materials

The following supporting information can be downloaded at: https://www.mdpi.com/article/10.3390/metabo12040281/s1, Figure S1: Examples of results from the correlation cluster.
