# Supplementary material for: Serum Metabolites Associated with Blood Pressure in Chronic Kidney Disease Patients
Source: Metabolites. 2022 Mar 23;12(4):281. doi: 10.3390/metabo12040281 (PMC9027690; doi:10.3390/metabo12040281)
Supplement: Supplementary file 1 [file metabolites-12-00281-s001.zip › metabolites-1605796-supplementary.pdf]

Supplementary Figures

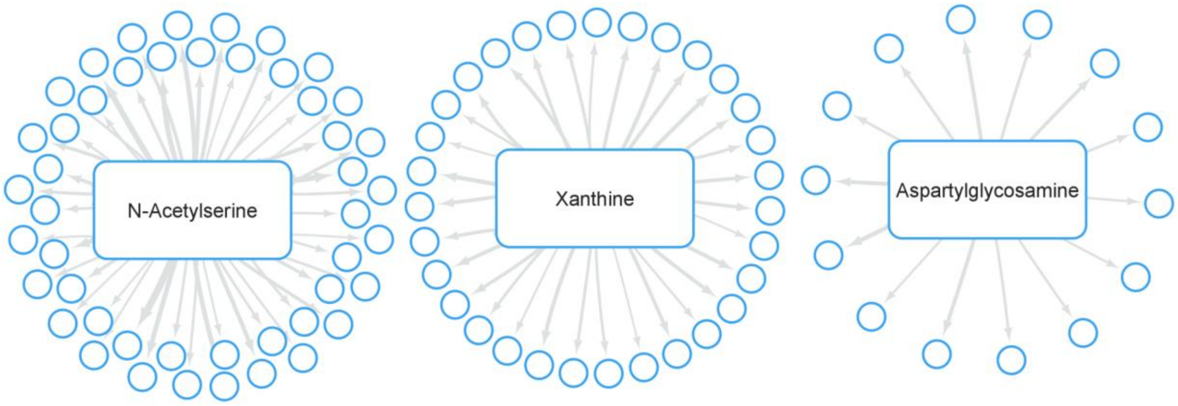

Supplementary Figure S1. Example results from feature reduction by correlation clustering algorithm.
